# The High-Affinity Potassium Transporter EpHKT1;2 From the Extremophile *Eutrema parvula* Mediates Salt Tolerance

**DOI:** 10.3389/fpls.2018.01108

**Published:** 2018-07-30

**Authors:** Akhtar Ali, Irfan Ullah Khan, Masood Jan, Haris Ali Khan, Shah Hussain, Muhammad Nisar, Woo Sik Chung, Dae-Jin Yun

**Affiliations:** ^1^Department of Biomedical Science and Engineering, Konkuk University, Seoul, South Korea; ^2^Division of Applied Life Science (BK21plus program), Gyeongsang National University, Jinju, South Korea; ^3^Department of Botany, University of Malakand, Chakdara, Pakistan

**Keywords:** *Arabidopsis*, *Eutrema parvula*, HKT1, Na^+^/K^+^ transporter, salt tolerance, glycophyte, halophyte

## Abstract

To survive salt stress, plants must maintain a balance between sodium and potassium ions. High-affinity potassium transporters (HKTs) play a key role in reducing Na^+^ toxicity through K^+^ uptake. *Eutrema parvula* (formerly known as *Thellungiella parvula*), a halophyte closely related to *Arabidopsis*, has two *HKT1* genes that encode EpHKT1;1 and EpHKT1;2. In response to high salinity, the *EpHKT1;2* transcript level increased rapidly; by contrast, the *EpHKT1;1* transcript increased more slowly in response to salt treatment. Yeast cells expressing EpHKT1;2 were able to tolerate high concentrations of NaCl, whereas EpHKT1;1-expressing yeast cells remained sensitive to NaCl. Amino acid sequence alignment with other plant HKTs showed that EpHKT1;1 contains an asparagine residue (Asn-213) in the second pore-loop domain, but EpHKT1;2 contains an aspartic acid residue (Asp-205) at the same position. Yeast cells expressing EpHKT1;1, in which Asn-213 was substituted with Asp, were able to tolerate high concentrations of NaCl. In contrast, substitution of Asp-205 by Asn in EpHKT1;2 did not enhance salt tolerance and rather resulted in a similar function to that of AtHKT1 (Na^+^ influx but no K^+^ influx), indicating that the presence of Asn or Asp determines the mode of cation selectivity of the HKT1-type transporters. Moreover, *Arabidopsis* plants (Col-*gl*) overexpressing *EpHKT1;2* showed significantly higher tolerance to salt stress and accumulated less Na^+^ and more K^+^ compared to those overexpressing *EpHKT1;1* or *AtHKT1*. Taken together, these results suggest that EpHKT1;2 mediates tolerance to Na^+^ ion toxicity in *E. parvula* and is a major contributor to its halophytic nature.

## Introduction

Soil salinity is a major abiotic stress that reduces crop productivity and yields (Huang et al., 2008). In most plants, saline soils lead to cytosolic osmotic stress and Na^+^ toxicity (Blumwald, 2000; Munns and Tester, 2008). Accumulation of high amounts of Na^+^ in the cytosol inhibits many processes such as protein synthesis, enzymatic reactions, and photosynthesis (Murguía et al., 1995; Tsugane et al., 1999). Plants use a number of sodium transporters to maintain sodium homeostasis sodium transporters, the plasma membrane Na^+^/H^+^ exchanger SOS1 extrudes excess Na^+^ from the cell via the Salt Overly Sensitive (SOS) pathway (Qiu et al., 2002; Quintero et al., 2002; Oh et al., 2009). Another class of Na^+^ transporters, high-affinity potassium transporters (HKTs; HKT1-type transporters), retrieve Na^+^ from the xylem stream and retain it in the roots, thus protecting the aerial tissues from damage (Rubio et al., 1995; Rus et al., 2001, 2006; Ren et al., 2005; Munns et al., 2012). The role of HKT1 transporters under salt stress has been well characterized in plants (Rus et al., 2001; Maser et al., 2002a; Berthomieu et al., 2003; Jha et al., 2010; An et al., 2017). HKT1-type transporters maintain a balance between sodium and potassium ions under salt stress to reduce sodium ion toxicity in the cell (Blumwald, 2000; Jha et al., 2010; Yao et al., 2010; Ali et al., 2012).

Some plants which are extremely tolerant to salt stress and use specialized mechanisms to survive in high-salinity environments are known as halophytes (Inan et al., 2004; Vinocur and Altman, 2005; Oh et al., 2014; Shao et al., 2014). Halophytes have a Na^+^ efflux system that distributes Na^+^ to various tissues and sequesters Na^+^ in the vacuole, thus reducing Na^+^ toxicity in sensitive tissues (Gong et al., 2005; Oh et al., 2009). The well-known halophytes *Eutrema salsuginea* and *Eutrema parvula* (formerly known as *Thellungiella halophila* and *Thellungiella parvula*, respectively) are closely related to *Arabidopsis* and are commonly used as model plants for studying salt stress (Inan et al., 2004; Oh et al., 2009; Ali et al., 2012). The genome of *E. salsuginea* has been sequenced and can be used to characterize the functions of different genes in the species (Wu et al., 2012).

HKT1 transporters are segregated into two subclasses, subclass1 and subclass2, based on their protein structure and ionic selectivity (Horie et al., 2001; Mäser et al., 2002b; Platten et al., 2006). The subclass1 transporters have a serine residue at the first pore-loop domain and show higher selectivity for Na^+^ than for K^+^, whereas the subclass2 transporters have a glycine residue at the same position and are considered to function as Na^+^/K^+^ co-transporters (Horie et al., 2001; Platten et al., 2006), although there are exceptions to this rule (Ali et al., 2016). Maintenance of Na^+^/K^+^ balance under salt stress is normally regulated by members of subclass2.

Although closely related, *Arabidopsis* and *Eutrema* have different numbers of HKTs. *Arabidopsis* has a single *HKT1* gene, *AtHKT1*, which codes for a subclass1-type transporter (Uozumi et al., 2000). AtHKT1 was found to highly specific for Na^+^ influx when expressed in *Xenopus laevis* oocytes and *Saccharomyces cerevisiae* (Uozumi et al., 2000). By contrast, *E. salsuginea* has three *HKT1* genes, *EsHKT1;1*, *EsHKT1;2*, and *EsHKT1;3*; each coding for a subclass1 HKT1transporter (Wu et al., 2012; Ali et al., 2016). The expression of *EsHKT1;2* is greatly induced under high salinity, but expression of *EsHKT1;1* is downregulated under salt stress, similar to AtHKT1 in *Arabidopsis* (Oh et al., 2010; Ali et al., 2012; Wu et al., 2012). When expressed in yeast and *X. laevis* oocytes, EsHKT1;2 showed a higher affinity for potassium than for sodium, whereas EsHKT1;1 showed a higher affinity for sodium than for potassium (Ali et al., 2016). *E. parvula* has two *HKT1* genes, *EpHKT1;1* and *EpHKT1;2*, that also code for subclass1 HKT1 transporters (Dassanayake et al., 2011).

Examination of the amino acid sequences of these HKT1s (three from *E. salsuginea* and two from *E. parvula*) showed that they contain a serine residue at the selectivity filter in the first pore-loop domain, and therefore are classified as subclass1 transporters (Ali et al., 2012). Subclass1 transporters are thought to be specific for Na^+^ transport, but EsHKT1;2 is an exception because it has higher affinity for K^+^ than for Na^+^ (Platten et al., 2006; Ali et al., 2016). Alignment of the amino acid sequences of all known HKTs with the yeast K^+^ transporter ScTRK1 provided additional clues about possible functional differences between HKT1 transporters (Ko and Gaber, 1991; Ali et al., 2012). EsHKT1;2 and EpHKT1;2 contain conserved aspartic acid (D) residues in their second pore-loop domain and also in the nearby transmembrane domain. In most HKT1 homologs in other species, this amino acid is an asparagine (Asn, N); however, yeast ScTRK1, which is an HKT, also carries an Asp residue in the pore-loop domain position (Ali et al., 2012). These reports suggest that the D/N dichotomy in this position is important for the embodiment of HKTs in subclass1 HKTs. Previously, we showed that single-residue substitutions at the D/N variance in the pore-loop domain inhibited the K^+^ uptake function of EsHKT1;2 and the Na^+^ uptake function of AtHKT1 (Ali et al., 2016). Thus, the cation selectivity of EsHKT1;2 and AtHKT1 is conferred by the specific amino acid residue at this position in the second pore-loop domain of the transporters. By contrast, there is no such report to explain the role of HKT1 homologs in *E. parvula*, an emerging extremophile model plant.

We report here that EpHKT1;1 and EpHKT1;2 from the extremophile *E. parvula* have different functions under salinity stress. In a yeast system, EsHKT1;2- and EpHKT1;2-expressing cells were able to tolerate better NaCl stress and the addition of potassium to the system further enhanced their resistance to NaCl. In contrast, EpHKT1;1-expressing cells were as sensitive to NaCl as cells expressing EsHKT1;1 and EsHKT1;3. The difference in the affinity toward K^+^ or Na^+^ between the different transporters was associated with the presence of conserved amino acids (D/N) in the second pore-loop domain. Furthermore, transgenic *Arabidopsis* plants overexpressing *EpHKT1;2* were tolerant to salt stress compared to those expressing *EpHKT1;1* or *AtHKT1*, indicating that EpHKT1;2 contributes to salt tolerance in *E. parvula*. Taken together, these results suggested that in addition to serine/glycine, the cation selectivity of HKTs can be determined by the presence aspartic acid/asparagine residues, in their second pore-loop domain.

## Materials and Methods

### Plant Material

Wild-type (WT) *Arabidopsis* seeds of Col-*gl1* and *35S::AtHKT1* (Ali et al., 2016) were used for this study.

### Generation of Transgenic *Arabidopsis* Plants

The cDNAs of *EpHKT1;2* and *EpHKT1-1* were amplified and cloned into the *pDONR/Zeo* GATEWAY vector (Invitrogen, Carlsbad, CA, United States). These entry vectors were further subcloned into the destination vector, pGWB14, and transformed into Col-*gl1* plants using the *Agrobacterium*-mediated flower-dipping method. Primers used for cloning are listed in Supplementary Table S1. Transgenic plants were selected based on hygromycin resistance and confirmed with the primers listed in Supplementary Table S1. Lines showing 3:1 segregation ratios with resistance to hygromycin (43 mg/L) were selected and homozygous T3 plants showing similar transcript levels of *EpHKT1;1* and *EpHKT1;2* (Supplementary Figure S3) were used for experiments.

### Growth Responses of Transgenic Plants to Salt Stress

To test the growth responses of the plants to salt stress, seeds of transgenic plants expressing *35S::AtHKT1, 35S::EpHKT1;2*, or *35S::EpHKT1;1*, and Col-*gl1* plants were surface sterilized and grown on 0.5X Murashige and Skoog (MS) medium containing different concentrations of NaCl in a long-day (16 h day/8 h night) growth chamber with 130 μmol m^-2^s^-1^ light intensity at 22–24°C. Photographs were taken after 7 days. To test the growth responses of the mature plants to salt stress, seedlings were grown under the same growth conditions noted above but without the NaCl. Seven-days-old seedlings were transferred to soil and further grown for 14 days. Plants were then treated with 300 mM NaCl in water every other day for 2 weeks. Photographs were taken after the salt treatment. Fresh weights of the plants were measured immediately at the end of the salt treatment.

### RNA Extraction, RT-PCR, and qRT-PCR Analysis

RNA from 10-days-old Col-*gl1* and transgenic plants was extracted with the Qiagen RNeasy plant mini kit (Qiagen, MD, United States). RT-PCR (reverse transcription polymerase chain reaction) was carried out with 3 μg of total RNA using the Thermo Script RT-PCR System (Invitrogen, Carlsbad, CA, United States). The primers used in RT-PCR or real-time PCR are listed in Supplementary Table S1. The conditions of real-time PCR were as follows: 95°C for 5 min, 45 cycles of 95°C for 10 s and 60°C for 30 s, followed by 95°C for 10 s, 65°C for 5 s, and 95°C for 5 s.

### Gene Expression and Growth in Yeast

Yeast strain*AXT3K* (*ena1*::*HIS3*::*ena4*, _*nha1*::*LEU2*, _*nhx1*:: *KanMX4*; Quintero et al., 2002) was used in this study. The cDNAs of *AtHKT1*, *EsHKT1;1*, *EsHKT1;2*, *EsHKT1;3*, *EpHKT1;1*, *EpHKT1;2*, and *AtKAT1* were amplified with the primers listed in Supplementary Table S1 and cloned into the *pYES2* vector (Invitrogen, Carlsbad, United States) between the GAL1 promoter and the CYC1 terminator sequences. Yeast cells were transformed by the LiAc method, selected on –URA synthetic dropout (SD) media and subjected to growth on synthetic complete medium with the indicated concentration of sodium and potassium as shown by Ali et al. (2016).

### Site-Directed Mutagenesis of EpHKT1;1 and EpHKT1;2

Site-directed mutagenesis was conducted according to the method described in Ali et al. (2012). The asparagine residue in the second pore-loop domain (N213) in EpHKT1;1 was replaced by aspartic acid (D) and the aspartic acid residue (D205) in EpHKT1;2 was replaced by asparagine (N) using the primers listed in Supplementary Table S1. Newly synthesized PCR products were treated with DPN1 enzyme and then transformed into *Escherichia coli*. Plasmids were extracted and sequenced for the targeted mutation.

### Analysis of Ion Content Plants

Ionic content analyses in plants were carried out as described (Rus et al., 2001) except that plants were grown for 2 weeks in 0.5X MS plates. Seedlings were treated with 100 mM NaCl for 12 and 24 h. Samples were dried at 65°C for 2 days and 100-mg ground tissue was extracted with 10 mL of 0.1 N HNO3 for 30 min. Samples were filtered and ion content analysis was carried out with inductively coupled plasma optical emission spectroscopy using an OPTIMA 4300DV/5300DV (Perkin-Elmer, Waltham, MA, United States).

### Sub-cellular Localization of EpHKT1;1 and EpHKT1;2

Full-length ORF sequences for both EpHKT1;1 and EpHKT1;2 were amplified with the primers listed in Supplementary Table S1, to generate entry vectors in the *pDONR^TM^/Zeo* vector (Invitrogen, Carlsbad, CA, United States). These entry vectors were further subcloned in destination vectors for sub-cellular localization assay. Both HKT1 proteins were fused in-frame to N-fragment of the eGFP fluorescent protein in the *pDEST-PK7WFG* vector. *Agrobacterium tumefaciens* strain GV3101 was transformed with the constructs. *Agrobacterium* grew in LB medium supplemented with 10 mM MES, 20 μM acetosyringone, and the appropriate antibiotics (dependent on the constructs used for transfection) and culture media were washed with infiltration solution (10 mM MgCl_2_, 10 mM MES, and 100 μM acetosyringone) twice to limit the toxicity of the antibiotics. *Agrobacterium* cells transformed with p19 silencing plasmid were included. For infiltration, each *Agrobacterium* culture was adjusted to OD_600_ 0.3 in final infiltration solution. The infiltrated leaves of 4-weeks-old Tobacco plants were incubated for 48–72 h, and then fluorescence was detected using a confocal laser scanning microscope (Olympus FV1000, Tokyo, Japan).

## Results

### The Tandem Duplication of *HKT1* in *E. salsuginea* and *E. parvula* Is Absent in *Arabidopsis*

**FIGURE 1:**
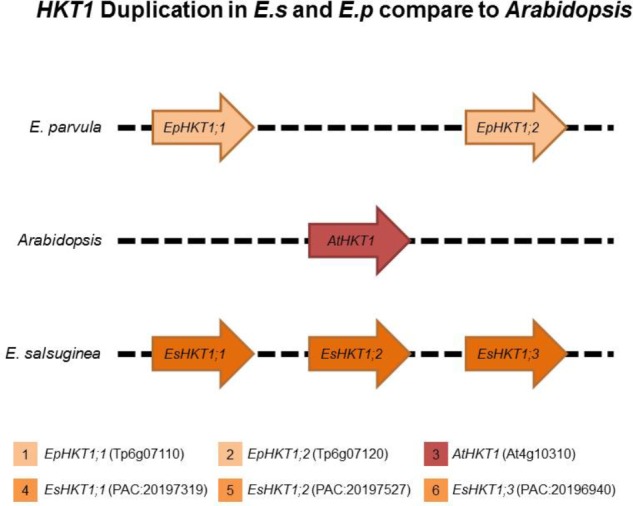
*HKT1* genes in *Arabidopsis thaliana*, *Eutrema parvula*, and *Eutrema salsuginea*. *Eutrema parvula* (Ep, also known as *Schrenkiella parvula*) and *E. salsuginea* (Es, previously known as *Thellungiella halophila* and *Thellungiella salsugineum*) are closely related to *Arabidopsis*. *HKT1* homologs are indicated as 1; (*EpHKT1;1*/Tp6g07120) and 2; (*EpHKT1;2*/Tp6g07110) located at EpChr6, 3; (*AtHKT1*/At4g10310) located at AtChr4 and 4; (*EsHKT1;1*/pacid = 20197527), 5; (*EsHKT1;2*/pacid = 20197319), and 6; (*EsHKT1;3*/pacid = 20196940) located at EsChr3. Different colored boxes indicate the *HKT1* genes in different species.

The *Arabidopsis thaliana* genome contains a single *HKT1* gene that codes for a sodium-selective transporter (Uozumi et al., 2000). *E. salsuginea* contains three *HKT1* genes and *E. parvula* contains two *HKT1* genes (Dassanayake et al., 2011; Wu et al., 2012; **Figure 1**). Based on suggested nomenclature and sequence similarities, all of these HKTs belong to the subclass1 HKT1 transporters (Platten et al., 2006).

Among the three HKT1s in *E. salsuginea*, EsHKT1;2 is the only transporter with a higher specificity for K^+^ than for Na^+^ under salt stress (Ali et al., 2016). We hypothesized that the *HKT1* gene was duplicated, followed by divergence of HKT function and the acquisition of K^+^ specificity in EsHKT1;2, a gene that is absent in *A. thaliana* and *Arabidopsis lyrata* (Ali et al., 2016). The same may be true for *E. parvula*, which contains two *HKT1* genes on chromosome 6, that code for EpHKT1;1 and EpHKT1;2 (**Figure 1**). Both of these proteins are localized to the plasma membrane, alike AtHKT1 (Supplementary Figure S1; Sunarpi et al., 2005).

### *EpHKT1;1* and *EpHKT1;2* Respond Differently to Salt Stress in *E. parvula*

**FIGURE 2:**
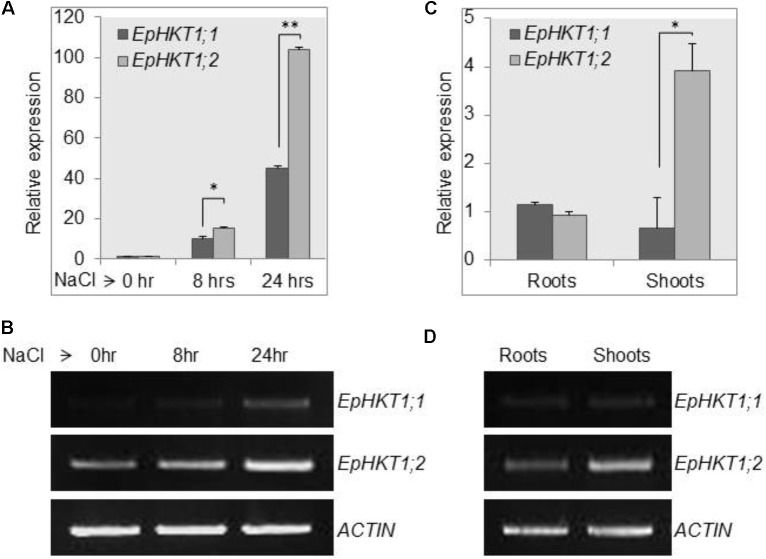
Upregulation of *EpHKT1;2* expression in response to salt stress. **(A,B)** The expression levels of *HKT1* genes under control and 150 mM salt-stress conditions were determined in 2-weeks-old *E. parvula* seedlings using quantitative **(A)** and semi-quantitative PCR **(B)** analysis. Error bars represent SE. Significant difference determined by Student’s *t*-test; (^∗^*P* < 0.05 and ^∗∗^*P* < 0.005). **(C,D)** Expression of *HKT1* genes in roots and shoots. For quantitative analysis, each sample was quantified at least in triplicate. *ACTIN2* was used as an internal control. Significant differences were determined by Student’s *t*-test; (^∗^*P* < 0.0001).

The expression of *EsHKT1;2* was rapidly induced in response to salt stress, thereby favoring K^+^ transportand Na^+^/K^+^ homeostasis (Ali et al., 2012; Wu et al., 2012). To test whether the *EpHKT1* genes are also regulated by salt stress in *E. parvula*, we examined the expression of *EpHKT1* genes under normal and salt-stress conditions. In a time-course experiment, expression of *EpHKT1;1* and *EpHKT1;2* was induced by NaCl in *E. parvula*; however, expression of *EpHKT1;2* was substantially higher compared with that of *EpHKT1;1* (**Figures 2A,B**). By contrast, a previous study showed that the level of *AtHKT1* transcripts in *Arabidopsis* declined during the same time periods under salt stress (Oh et al., 2010). *EpHKT1;2* transcripts were abundant in shoots, while similar levels of *EpHKT1;1*and *EpHKT1;2* were observed in roots (**Figures 2C,D**). The same pattern was observed for *EsHKT1;2* in *E. salsuginea* (Ali et al., 2012). Among the three known *HKT1* genes in *E. salsuginea*, *EsHKT1;2* was expressed more abundantly and also induced by salt stress, while expression of *EsHKT1;1* and *EsHKT1;3* remained much lower (Wu et al., 2012). *EsHKT1;2* expression is required for the halophytic behavior of *E. salsuginea* under salt stress (Ali et al., 2013). Therefore, we hypothesized that *EpHKT1;2* could also convey tolerance to Na^+^ ions in *E. parvula* due to its remarkable induction upon salt stress.

### Yeast Cells Expressing EpHKT1;2 and EsHKT1;2 Were Less Sensitive to High Levels of NaCl

**FIGURE 3:**
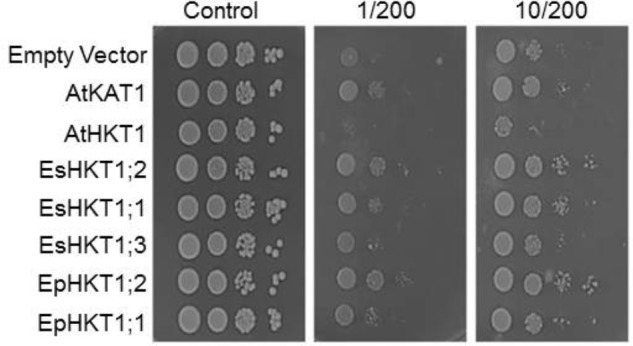
EpHKT1;2-expressing yeast cells were less sensitive to high NaCl. Yeast cells of strain AXT3K transformed with an empty vector (EV, negative control), or expressing AtKAT1 (positive control), AtHKT1, EsHKT1;1, EsHKT1;2, EsHKT1;3, EpHKT1;1, and EpHKT1;2 were grown overnight and serial decimal dilutions were spotted on SC dropout agar medium without uracil. Indicated concentrations of sodium (200 mM) and potassium (1 or 10 mM) were added to the medium. Photographs were taken after 3 days.

An earlier study using yeast cells indicated the importance of EsHKT1;2 (TsHKT1;2) for salt tolerance among the three homologs of HKT1 in *E. salsuginea*, and showed that EsHKT1;2 has stronger affinity for K^+^ than for Na^+^ (Ali et al., 2016). *EpHKT1;2* of *E. parvula* shows strong similarities to *EsHKT1;2* and their transcript are both highly upregulated in response to salt stress (**Figure 2**; Ali et al., 2012). Therefore, we expressed genes in the sodium-sensitive yeast strain AXT3K the two HKTs from *E. parvula*, the three HKTs from *E. salsuginea*, and AtHKT1 of *A. thaliana*. Yeast cells expressing EsHKT1;2 or EpHKT1;2 showed the same phenotype of relative tolerance to Na^+^ ions compared to all other HKTs (**Figure 3**). An increase in K^+^ concentration in the medium further enhanced the growth of all cells, including those expressing the K^+^-selective channel KAT1, and reduced the growth differences between strains (**Figure 3**). These data indicate that toxicity was due to Na^+^ influx and suppression of K^+^ uptake, and that cells expressing EsHKT1;2 and EpHKT1;2 had a selective advantage under K^+^ limitation (**Figure 3** and Supplementary Figure S2). The results strongly indicated that EsHKT1;2 and EpHKT1;2 have a higher affinity for K^+^, whereas EsHKT1;1, EsHKT1;3, and EpHKT1;1, like AtHKT1, showed a higher affinity for Na^+^. Yeast cells expressing EsHKT1;1, EsHKT1;3, or EpHKT1;1 grew slightly better than yeast cells expressing AtHKT1, indicating that additional amino acid differences, presently unknown, may further enhance K^+^ uptake or restrict Na^+^ permeation.

### The Cation Selectivity of EpHKT1;1 and EpHKT1;2 Is Associated With the Presence of Asparagine or Aspartic Acid in the Second Pore-Loop Region

**FIGURE 4:**
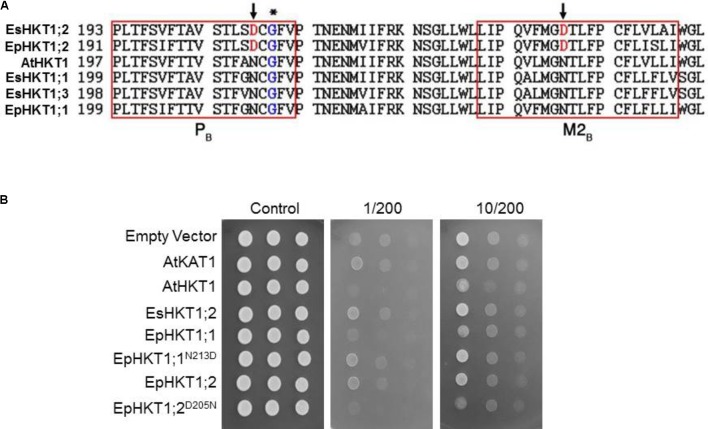
Presence of Asp (D) or Asn (N) in the second pore-loop region confers cation selectivity. **(A)** Comparison of HKT1 homologs from *Arabidopsis, E. salsuginea*, and *E. parvula*. Amino acid sequences in the second pore-loop region (P_B_) and the adjacent transmembrane domain (M2_B_) were aligned by ClustalW (http://www.ebi.ac.uk/Tools/msa/clustalw2/). The conserved glycine residues in the P_B_ region (Mäser et al., 2002b) are indicated by an asterisk. The aspartic acid residues specific for EsHKT1;2 and EpHKT1;2 are indicated by arrows. **(B)** Na^+^-induced growth inhibition of yeast strain AXT3K. Yeast cells transformed with an empty vector (EV, negative control), or expressing AtKAT1 (positive control), AtHKT1, EsHKT1;2, EpHKT1;1, EpHKT1;2, and the mutant forms of EpHKT1;1 (EpHKT1;1N213D) and EpHKT1;2 (EpHKT1;1D205N) were grown overnight and the serial dilutions were spotted on SC dropout agar medium without uracil. Indicated concentrations of sodium (200 mM) and potassium (1 or 10 mM) were added to the medium. Photographs were taken after 3 days.

AtHKT1 and EsHKT1;2 showed differences in their ionic selectivity in yeast lines and *Xenopus* oocytes based on the presence of Asp or Asn in key positions in these transporters (Ali et al., 2016). Alignment of their amino acid sequences showed that EpHKT1;2 has an Asp205 residue in the second pore-loop domain, which was replaced by an Asn213 residue in EpHKT1;1 (**Figure 4A**). The presence of Asp (D) in the second pore-loop domain of EpHKT1;2 and EsHKT1;2 is not conserved among all other known HKTs that have Asn (N) residues at the equivalent position (**Figure 4A**, Ali et al., 2016). To test whether the presence of Asp205 and Asn213 is responsible for the differences in cation specificity between EpHKT1;2 and EpHKT1;1, we replaced Asp205 in EpHKT1;2 with asparagine (D205N), and Asn213 in EpHKT1;1 with aspartic acid (N213D; **Figure 4B**). When expressed in yeast (AXT3K), these mutant proteins showed changes in their cation preference (**Figure 4B**). Compared to EpHKT1;2, high concentrations of Na^+^ resulted in reduced growth in yeast cells expressing EpHKT1;2^D205N^, whereas yeast cells expressing EpHKT1;1^N213D^ showed enhanced Na^+^ tolerance compared to EpHKT1;1 (**Figure 4B**). Taken together, these results suggested that the presence of asparagine or aspartic acid in the second pore-loop region in HKT1-type transporters determines their cation specificity (Na^+^ or K^+^).

### *EpHKT1;2*-Overexpressing Plants Are Tolerant to Salt Stress

**FIGURE 5:**
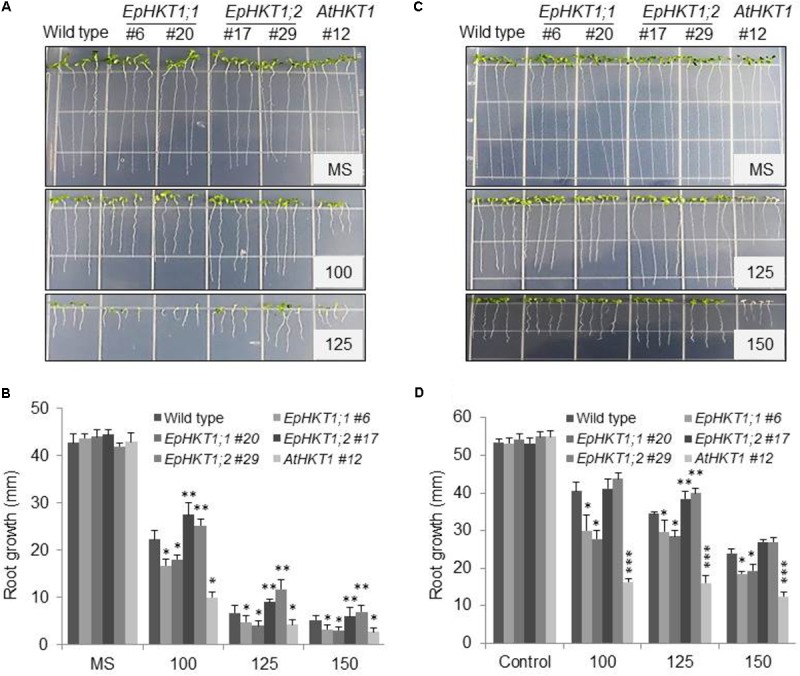
*EpHKT1;2*-overexpressing *Arabidopsis* plants are salt tolerant compared to *EpHKT1;1*- or *AtHKT1*-overexpressing plants. **(A)** Seeds of the wild type (Col-*gl1*) and indicated transgenic lines were germinated on 1XMS medium with or without the indicated concentrations of NaCl (mM) in a growth chamber under long-day conditions (16 h light, 8 h dark) and grown vertically for 1 week. Photographs were taken 1 week after germination. **(B)** Root growth of the plants grown under different concentration of NaCl. Plates were put vertically in a growth chamber under long-day conditions. Photographs were taken 7 days after germination. Error bars represent SE. Significant differences were determined by Student’s *t*-test (^∗^*P* < 0.005 and ^∗∗^*P* < 0.05). **(C)** Seeds of the wild type (Col-*gl1*) and indicated transgenic lines were germinated on 1XMS plates, allowed to grow for 4 days and then transferred to new plates containing 1X MS medium with different concentrations of NaCl. Photographs were taken after 1 week under NaCl stress. **(D)** Root growth of the 4 days transferred plants that were further allowed to grow for further 7 days under different concentrations of NaCl. Plates were put vertically in a growth chamber under long-day conditions. Photographs were taken 7 days after transfer to NaCl plates. Error bars represent SE. Significant differences were determined by Student’s *t*-test (^∗^*P* < 0.05, ^∗∗^*P* < 0.005, and ^∗∗∗^*P* < 0.001) compared with Col-*gl1* under salt treatment.

To investigate whether the HKTs in *E. parvula* might contribute to its halophytic nature, *EpHKT1;1*, *EpHKT1;2*, and *AtHKT1;1* were ectopically expressed in *Arabidopsis* (Col-*gl1*) under the control of the *Cauliflower mosaic virus* (CaMV) 35S promoter. Several homozygous transgenic lines with similar expression levels of the *HKT1*geneswere selected (Supplementary Figure S3). Seeds of Col-*gl1* and the transgenic lines were grown on 1XMS media containing different concentrations of NaCl for up to 7 days. Consistent with previous findings, *AtHKT1*-overexpressing plants were hypersensitive to salt stress and showed severe reductions in root growth (**Figures 5A,B**; Møller et al., 2009). *Arabidopsis* plants expressing *EpHKT1;1* also showed reductions in root growth but were less sensitive to salt stress compared to those expressing *AtHKT1* (**Figures 5A,B**). By contrast, plants expressing *EpHKT1;2* were more tolerant to salt stress compared to all other tested lines (**Figures 5A,B**), indicating that EpHKT1;2 has a different function than the other tested HKTs (**Figures 5A,B**).

### Over-Expression of *EpHKT1;2* in *Arabidopsis* Plants Conferred Greater Tolerance to Salt Stress Compared to Its Homolog *EpHKT1;1*

**FIGURE 6:**
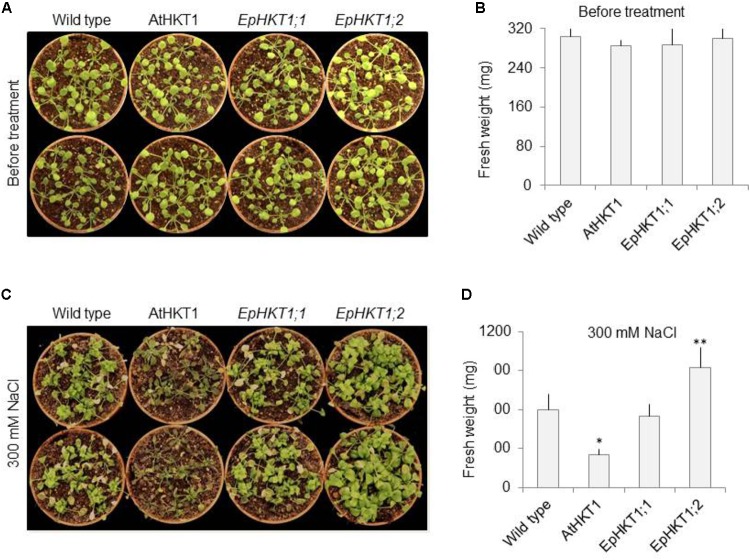
Salt-stress responses of soil-grown *EpHKT1;1*- and *EpHKT1;2*-overexpressing plants compared with *AtHKT1*-overexpressing plants. **(A)** Seeds were germinated on 1XMS medium in a growth chamber under long-day conditions (16 h light, 8 h dark). One-week-old seedlings were then transferred to soil and grown for 2 more weeks. Photographs were taken before salt treatment. **(B)** Fresh weight of plants shown in **A**. **(C)** Two-weeks-old plants, as shown in A, were treated with 300 mM NaCl twice a week for 2 weeks. Photographs were taken at the end of salt treatment. **(D)** Fresh weight of plants shown in **C**. Error bars represent SE. Significant differences were determined by Student’s *t*-test (^∗^*P* < 0.001 and ^∗∗^*P* < 0.01) compared with Col-*gl1* under salt treatment.

**FIGURE 7:**
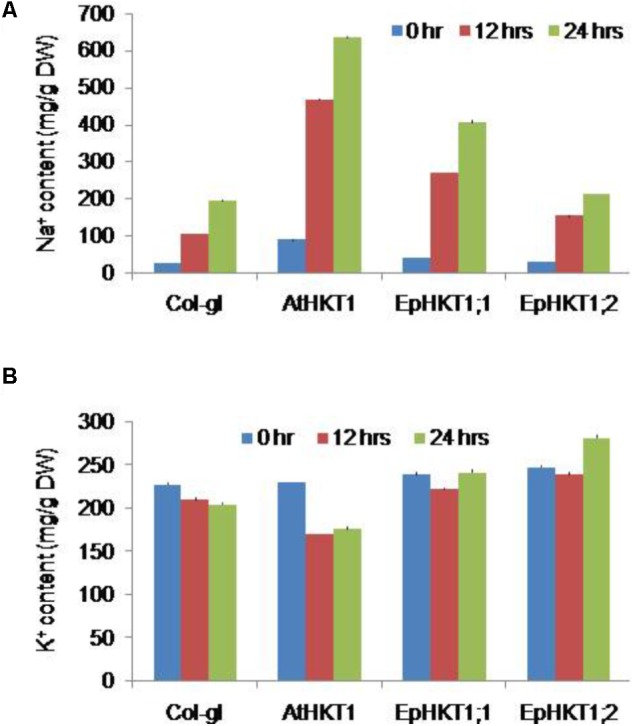
Na^+^ and K^+^ content in plants. Two-weeks-old seedlings of Col-*gl1* and transgenic plants expressing *35S::AtHKT1*, *35S::EpHKT1;1*, or *35S::EpHKT1;2* in the Col-*gl1* background were treated with 100 mM NaCl for 12 and 24 h in MS medium. Na^+^ **(A)** and K^+^ **(B)** contents were measured by inductively coupled plasma optical emission spectroscopy.

Next, we investigated the salt stress phenotypes of the transgenic lines in soil. Under normal growth conditions, all lines showed a similar healthy growth and comparable fresh weights (**Figures 6A,B**). When exposed to 300 mM NaCl, *EpHKT1;1*-overexpressing plants showed a decrease in growth comparable to the WT, whereas *AtHKT1*-overexpressing plants showed severe salt sensitivity (**Figure 6C**). By contrast, the plants expressing *EpHKT1;2* were more tolerant to salt stress and accumulated more fresh weight compared to all other tested lines including the WT (**Figures 6C,D**).The superior performance of *EpHKT1;2* plants becomes more evident as the period of salt stress was extended to 3 weeks (Supplementary Figure S4). In addition, *EpHKT1;2*-expressing plants accumulated less Na^+^ and more K^+^ than those expressing *EpHKT1;1* or *AtHKT1*, which strongly support their salt-tolerant phenotypes (**Figure 7**). These results are consistent with the previous finding that EsHKT1;2 mediates improved K^+^/Na^+^ balance under high salinity, which in turn reduces Na^+^ toxicity (Ali et al., 2016). Our results support a critical role of EpHKT1;2 in the salt tolerance of *E. parvula*.

## Discussion

HKT1-type transporters play a crucial role in plant adaptation to salt stress. HKT1-type transporters mediate the distribution of Na^+^ within the plant by removing Na^+^ from the xylem, particularly in the roots, to reduce Na^+^ toxicity in the shoots (Munns and Tester, 2008; Hamamoto et al., 2015). HKT1 homologs have been identified in a number of plant species, including *Arabidopsis*, and their ion selectivity characterized in yeast and *Xenopus* oocytes (Rubio et al., 1999; Fairbairn et al., 2000; Uozumi et al., 2000; Golldack et al., 2002; Garciadeblás et al., 2003; Su et al., 2003; Haro et al., 2005; Takahashi et al., 2007; Jabnoune et al., 2009; Asins et al., 2012; Shao et al., 2014; Wang et al., 2014; Ali et al., 2016). HKT1-type transporters are classified into two subclasses, subclass1 and subclass2, based on their protein structure and ion selectivity (Platten et al., 2006). All HKT1s from dicots contain a serine residue at the predicted filter in the pore-loop A domain. Like AtHKT1, EsHKT1;2 (formerly TsHKT1;2) and EpHKT1;2 belong to subclass1 and contain the conserved serine residue in the selectivity filter (Ali et al., 2012). However, the behavior of EsHKT1;2 and EpHKT1;2, which show significantly higher affinity for potassium than for sodium ions, differs from the other subclass 1 proteins (**Figures 4**, **7**; Ali et al., 2016).

Potassium uptake is important for plants during salt stress (Qi and Spalding, 2004; Anschütz et al., 2014). High Na^+^ content in the cytosol leads to severe K^+^ deficiency. One strategy to counteract K^+^ deficiency is to activate high-affinity K^+^ transporters to take up K^+^ and thus maintain the ionic balance at the cell level (Maathuis and Amtmann, 1999). Salt stress leads to upregulation of *EsHKT1;2* and *EpHKT1;2*, suggesting their role in salinity stress (**Figure 2**; Wu et al., 2012). This pattern of upregulation is different from that of *AtHKT1* in *Arabidopsis*, which imports Na^+^ instead of K^+^ under salinity stress (Uozumi et al., 2000; Ali et al., 2012). EpHKT1;2 and EsHKT1;2 will be instrumental in the capture and redistribution of K^+^ in *E. parvula* and *E. salsuginea*, respectively, based on their transcriptional activation in response to high salinity and their permeability to K^+^ (**Figures 2**, **3**; Ali et al., 2016).

Protein sequence alignment showed that all HKTs from *E. parvula* and *E. salsuginea* have high similarity to AtHKT1 (**Figure 4A**). However, AtHKT1 functions as a selective Na^+^ transporter in yeast and *X. laevis* oocytes (Uozumi et al., 2000), but EsHKT1;2 and EpHKT1;2 function as K^+^ transporters with lower affinity toward Na^+^ (**Figure 4B**; Ali et al., 2016). Therefore, although they are categorized as subclass1 proteins, EsHKT1;2 and EpHKT1;2 do not behave the same as other HKT1 proteins in this subclass (**Figure 3**; Ali et al., 2012).

High-affinity K^+^ transporters, as well as Na^+^/H^+^ antiporters, are activated during salt stress (Oh et al., 2009). Other transporters are also involved in partitioning Na^+^ into the vacuole, which can act as the ultimate sink for Na^+^ ions (Kronzucker and Britto, 2011). The localization of AtHKT1 in xylem parenchyma cells relates to its role in reducing the flux of sodium ions to the shoot tip in the presence of excess Na^+^. For plants such as halophytes that may be exposed to potentially toxic levels of Na^+^, the function of some HKT1 isoforms seems to have changed from excluding Na^+^ flux throughout the plant into functioning as K^+^ transporters. The ability of *Eutrema* species to maintain a low cytosolic Na^+^/K^+^ ratio in the presence of high salinity has been shown (Orsini et al., 2010). Downregulation of *EsHKT1;2* by RNA interference (RNAi) leads to a hyper-accumulation of Na^+^ in the shoots and lower concentrations in the roots compared to the WT, indicating that EsHKT1;2 in *E. salsuginea* regulates Na^+^ uptake under salt stress. The Na^+^/K^+^ ratio was also disturbed in *EsHKT1;2RNAi* lines (Ali et al., 2012). We hypothesized that EpHKT1;2, like EsHKT1;2, contributes to salt tolerance based on its ectopic expression in *Arabidopsis* (**Figures 6**, **7**). *EpHKT1;2*-overexpressing *Arabidopsis* plants were more tolerant to salt stress than all other tested plants (**Figure 6**). In contrast, *EpHKT1;1*-overexpressing *Arabidopsis* plants were sensitive to salt stress, but plants overexpressing *AtHKT1* were the most sensitive to salt stress (**Figures 5**, **6**). Analysis of ion contents in transgenic lines also showed enhanced retention of K^+^ and reduced Na^+^ content in *Arabidopsis* plants overexpressing *EpHKT1;2*, which is consistent with reduced Na^+^ transport and toxicity (**Figure 7**; Asins et al., 2012).

These results agree with previous findings on the modeled structures generated for AtHKT1 and EsHKT1;2 showing dissimilar charge distributions at the pore-loop domain (Ali et al., 2016). Aspartate residues in the pore-forming region created a strong negatively charged surface to which K^+^ ions were attracted as the result of a strong salt-bridge interaction between the K^+^ ion and oxygen atoms in Asp residues, thereby favoring selective K^+^ permeation through the EsHKT1;2 transporter. The presence of conserved amino acids (Asp and Asn) in the pore-loop region of HKT1 variants from plant species with contrasting salt tolerance underscores their importance in cation selectivity. In this work, we differentiated EpHKT1;2 from EpHKT1;1 based on the amino acid sequence and the pore domain and their ionic selectivity. EpHKT1;2 contains an Asp (D) residue and EpHKT1;1 contains an Asn (N) residue in their selectivity filter positions (**Figure 4A**). Substitution of these amino acids in the two proteins altered their functions: the addition of Asp residue conferred salt tolerance to yeast expressing mutated EpHKT1;1 (**Figure 4B**), while its WT form was associated with salt sensitivity in yeast and plants (**Figures 4**–**7**).

## Conclusion

Expressing *EpHKT1;2* or *EsHKT1;2* in *Arabidopsis* cells and tissues that normally express *AtHKT1* led to an increase in K^+^ content, which counteracted the deleterious effect of high concentrations of Na^+^ in the media.

## Author Contributions

AA and D-JY conceived and designed the experiments. AA, MJ, HK, and SH performed the experiments. AA, MN, WC, and D-JY analyzed the data. AA, IK, and D-JY wrote the paper. All authors reviewed and approved the final manuscript.

## Conflict of Interest Statement

The authors declare that the research was conducted in the absence of any commercial or financial relationships that could be construed as a potential conflict of interest.
