# Supplementary material for: The High-Affinity Potassium Transporter EpHKT1;2 From the Extremophile Eutrema parvula Mediates Salt Tolerance
Source: Front Plant Sci. 2018 Jul 30;9:1108. doi: 10.3389/fpls.2018.01108 (PMC6077265; doi:10.3389/fpls.2018.01108)
Supplement: Supplementary file 1 [file Presentation_1.pdf]

Supplemental Information

Supplemental Figures.

Supplemental Figure 1.

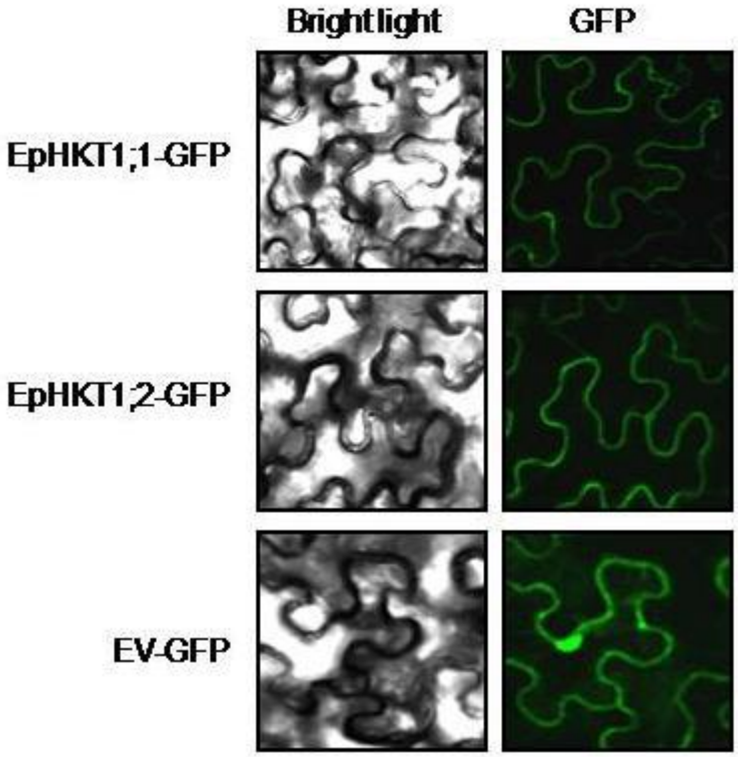

Figure S1. EpHKT1;1 and EpHKT1;2, both are localized to plasma membrane. GFP-tagged EpHKT1;1 and EpHKT1;2 were agro-infiltrated in tobacco leaf epidermal cells. After 3days infiltrated leaves were analyzed by confocal microscopy.

Supplemental Figure 2.

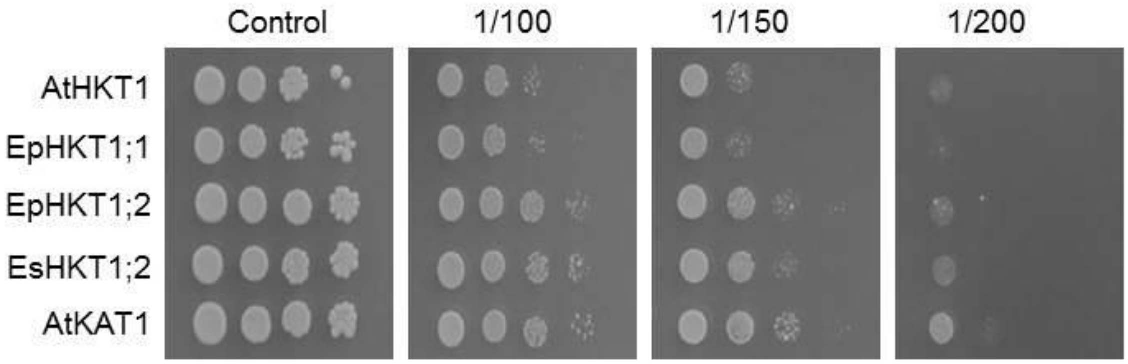

Figure S2. EpHKT1;2-expressing yeast cells tolerate NaCl stress. Yeast cells of strain AXT3K expressing AtHKT1, EpHKT1;1, EpHKT1;2, EsHKT1;2, and AtKAT1 (as a positive control for K+ transport), were grown overnight and serial decimal dilutions were spotted on SC dropout agar medium without uracil. Indicated concentrations of sodium and potassium were added to the medium. Photographs were taken after 3days.

Supplemental Figure 3.

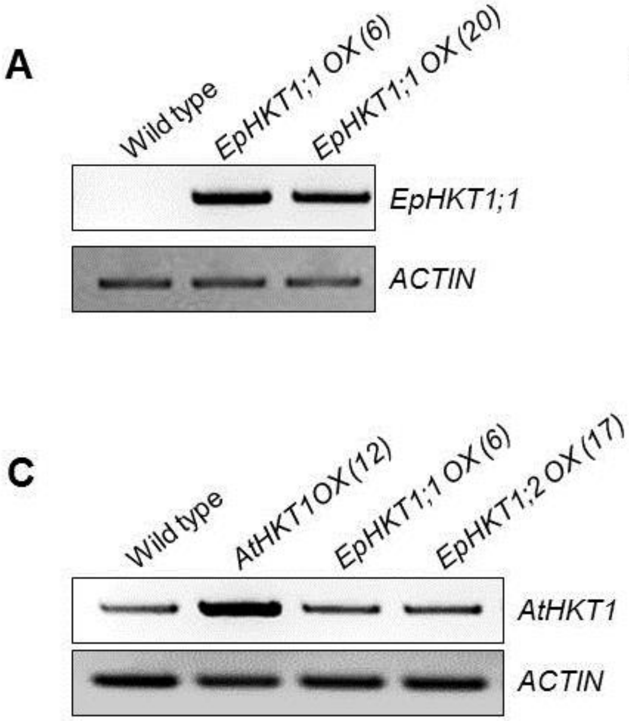

Figure S3. Semi-quantitative RT-PCR of selected transgenic lines with similar levels of ectopic *HKT1* expression. Transgenic *Arabidopsis* lines overexpressing either *EpHKT1;1* **(A)**, *EpHKT1;2* **(B)**, or *AtHKT1* **(C)**, under the control of the *Cauliflower mosaic virus* (CaMV) 35S promoter with similar levels of transgene expression were selected. Primers used are listed in **Table S1**.

Supplemental Figure 4.

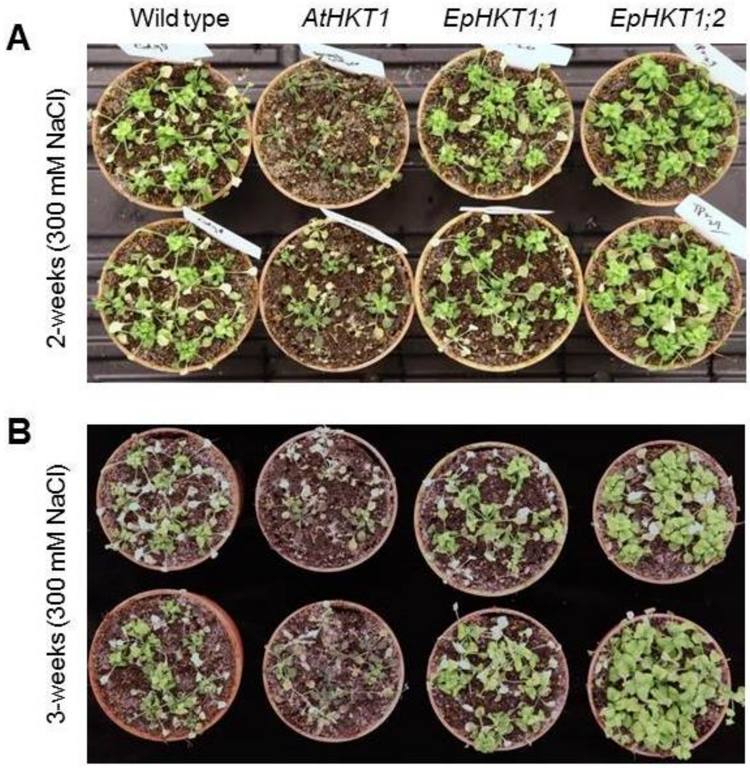

Figure S4. *EpHKT1;2*-expressing plants are more tolerant to salt stress than those expressing *EpHKT1;1* or *AtHKT1*. (A) Seeds of indicated lines were germinated on 1XMS medium in a growth chamber under long-day conditions (16h light, 8h dark). One-week-old seedlings were transferred to soil and further grown for 2weeks. For the saline treatment, pots were immersed twice a week in 300 mM NaCl for 2weeks. Photographs were taken at the end of salt treatment. **(B)** Plants shown in **(A)** were further grown for 1week before photographs were taken.

Supplemental table S1. PCR primer sequences used for this article

Primer name	Sequence (5'-3')
pYES2-AtHKT1-For	GATCGGATCCATGGACAGAGTGGTGGCA
pYES2-AtHKT1-Rev	ATAAGAATGCGGCCGCTCAGGAAGACGAGGGGTAAAG
pYES2-EsHKT1;2-For	GATCGGATCCATGGAGAGAGTTGTGGAC
pYES2-EsHKT1;2-Rev	ATAAGAATGCGGCCGCTCACGAAGATGAAGGATAAAG
pYES2-EsHKT1;1-For	GATCGAATTCATGGAGAGAGTTGGGGCA
pYES2-EsHKT1;1-Rev	AGAATGCGGCCGCTCAGAAAGAGGAGGGATAAAGAATC
pYES2-EsHKT1;3-For	GATCGGATCCATGGAGAGAATTGATGCAAAATTCGCTAAACTCGGTTCCC
pYES2-EsHKT1;3-Rev	ATAAGAATGCGGCCGCTCACGAAGAGGAGGGATAAAGTATCCA
pYES2-EpHKT1;1-For	GATCGGATCCATGGAGAGAGTTGTGGCAAAATTAGCTAAACTTCG
pYES2-EpHKT1;1-Rev	ATAAGAATGCGGCCGCTTATGGATAAAGTATCCATGCTCGGCCAGA
pYES2-EpHKT1;2-For	GATCGGATCCATGGAGAGAGTTGTAGCAAAATTAGCTAG
pYES2-EpHKT1;2-Rev	ATAAGAATGCGGCCGCTTAGTAAGAAGAGGATGGATAAAGTATCCAC
pYES2-AtKAT1-For	GATCGGATCCATGTCGATCTCTTGGACTCG
pYES2-AtKAT1-Rev	ATAAGAATGCGGCCGCTCAATTTGATGAAAAATAC
pDON-EpHKT1;1-For	AAAAAAGCAGGCTTCATGGAGAGAGTTGTGGCAAAATTAGCTAAA
pDON-EpHKT1;1-Rev	AGAAAGCTGGGTCTTATGGATAAAGTATCCATGCTCGGCC
pDON-EpHKT1;2-For	AAAAAAGCAGGCTTCATGGAGAGAGTTGTAGCAAAATTAGCT
pDON-EpHKT1;2-Rev	AGAAAGCTGGGTCTTAGTAAGAAGAGGATGGATAAAG
pDON-AtHKT1-For	AAAAAAGCAGGCTTCATGGACAGAGTGGTGGCA
pDON-AtHKT1-Rev	AGAAAGCTGGGTCTCAGGAAGACGAGGGGTAAAG
qRT-EpHKT1;1-For	AAACTTCGTTCGCAACTTGCTAA

qRT-EpHKT1;1-Rev	TGTCGACGGTGGACATGGAAGACG
RT-EpHKT1;1-Rev	GATCCTCAGTTGGACGCTTT (Use qRT-EpHKT1;1-For for pair)
qRT-EpHKT1;2-For	AGCAAAATTAGCTAGATC
qRT-EpHKT1;2-Rev	ATGTCGACGGTGGACATGGAAGAGA
RT-EpHKT1;2-Rev	CCGATCATCGATCGAAGTGTCC (Use qRT-EpHKT1;1-For for pair)
RT-AtHKT1-For	TCTTCTTGGAGTGACGGTGC
RT-AtHKT1-Rev	ACGTTGAAATTTATCGGATC
EpHKT1;1N213D-For	TCCACGTTCGGAGACTGTGGATTTGTC
EpHKT1;1N213D-Rev	GACAAATCCACAGTCTCCGAACGTGGA
EpHKT1;2D205N-For	TCTACGTTATCAAACTGTGGATTTGTC
EpHKT1;2D205N-Rev	GACAAATCCACAGTTTGATAACGTAGA
